# Horner’s Syndrome and Vertebral Artery Occlusion Concomitant With Brachial Plexus Injury in a Patient With Anterior Approach Cervical Disc Herniation Surgery

**DOI:** 10.7759/cureus.17810

**Published:** 2021-09-08

**Authors:** Seniz Akcay, Ali Murat Koc, Neslihan Eskut, Asli Koskderelioglu

**Affiliations:** 1 Physical Medicine and Rehabilitation, University of Health Sciences, Izmir Bozyaka Education and Research Hospital, Izmir, TUR; 2 Radiology, Izmir Katip Celebi University, Faculty of Medicine, Izmir, TUR; 3 Neurology, University of Health Sciences, Izmir Bozyaka Education and Research Hospital, Izmir, TUR

**Keywords:** cervical spine, horner’s syndrome, vertebral artery, brachial plexus injury, disc herniation surgery

## Abstract

Horner's syndrome is one of the rare complications after anterior approach intervertebral disc herniation surgery. Here, we described a 35-year-old male patient with Horner's syndrome accompanied by brachial plexus injury at the upper trunk level and vertebral artery occlusion after anterior ipsilateral approach cervical discectomy and cervical disc prosthesis operation. We are not aware of a similar case of these complications after this operation in the literature. After the six-month follow-up period the patient's Horner’s syndrome slightly improved and he partially gained right upper extremity muscle strength.

## Introduction

Lesion of the ipsilateral oculosympathetic pathway may result in Horner’s syndrome. Lower cervical and upper thoracic spinal cord lesions have shown to be responsible for Horner’s syndrome [1-4].

It has been reported as a rare complication of anterior approach cervical disc herniation surgery. The anterior approach enables a direct route to the transverse foramen with less retraction of the carotid sheath neurovascular complex. The prolonged retraction of longus colli muscle is reported to be the main cause of Horner’s syndrome that arises after the anterior approach disc herniation surgery [5,6].

We described a case presenting with Horner’s syndrome, vertebral artery (VA) occlusion, and brachial plexus injury developed soon after anterior approach cervical discectomy prosthesis surgery. Although Horner’s syndrome generally has a good prognosis, he improved slowly due to additional neurological impairment.

## Case presentation

A 35-year-old male patient was admitted to the outpatient clinic with right upper extremity weakness and numbness. He suffered from neck pain and numbness in the left arm for five years, but transient weakness occurred. He was consulted with neurosurgery with cervical magnetic resonance imaging (MRI) compatible with C5-6 left paramedian disc extrusion. Anterior approach cervical discectomy and cervical disc prosthesis operation with the right approach were performed. He presented with right ptosis, anhidrosis at the right part of his face, and right upper extremity weakness soon after the surgery.

**Figure 1:**
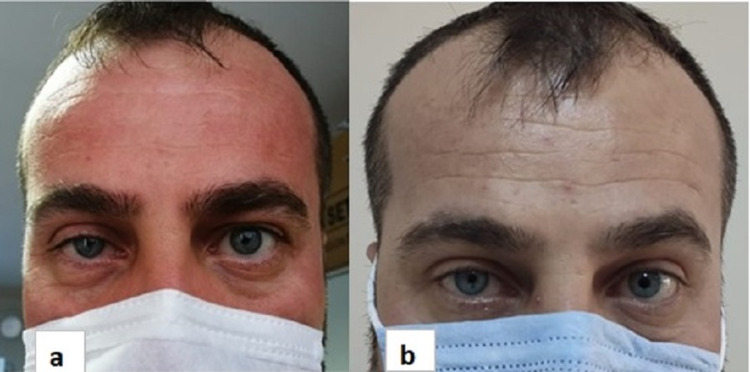
Right Horner’s syndrome Myosis and ptosis appearance of the patient one month (a) and six months after surgery (b).

We found right Horner’s syndrome (Figure 1). The light reflex of the pupil was preserved. Motor examination revealed shoulder abduction 2/5, shoulder external rotation 3/5, elbow flexion 3/5, and forearm supination 3/5 according to Medical Research Council standards. Light touch and pinprick tests were impaired at C5 and C6 dermatomes on the right side. Biceps and brachioradialis deep tendon reflexes were reduced on the right side. The rest of the neurological examination was normal.

**Figure 2:**
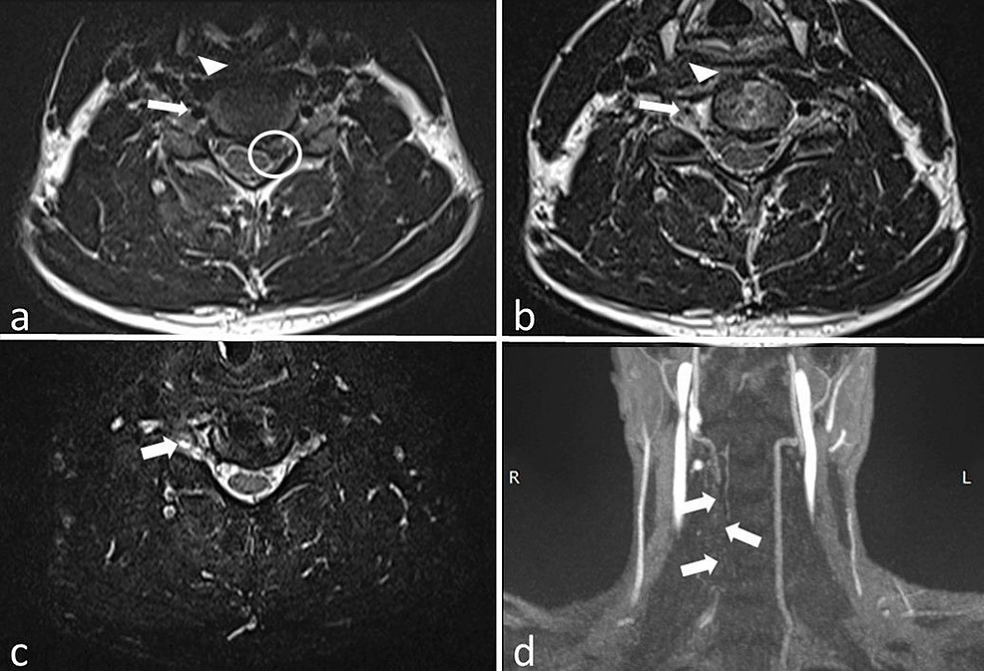
Cervical MR and MR Angiography Axial pre-(a) and post-(b) operative T2-weighted images demonstrate the change in the caliber of the right vertebral artery (arrows) along with the prominent atrophy in the right longus colli muscle (arrowheads). The preoperative extruded C5-6 intervertebral disk is also shown in the circle (a). Axial T2-space image (c) depicts a pseudomeningocele cavity at the post-foraminal segment of the right C5 nerve (arrow). Coronal MR angiography image (d) confirms the asymmetrical absence of flow in the right vertebral artery (arrows). MR, magnetic resonance.

**Figure 3:**
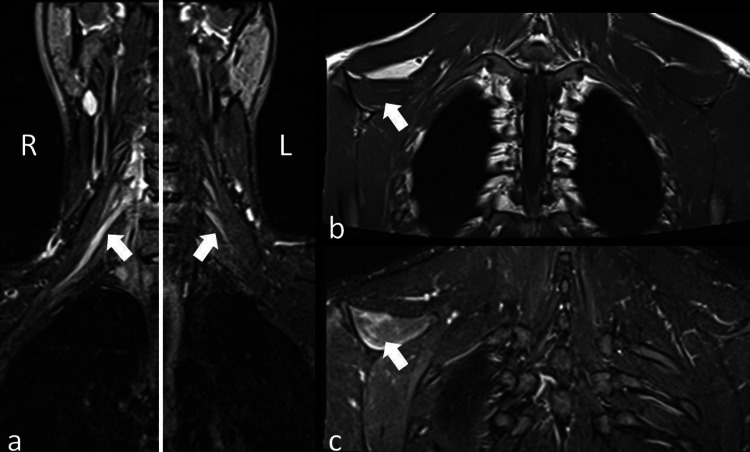
T2-TIRM MR image Coronal T2-TIRM MR image (a) with a fused view from both sides of the brachial plexus shows asymmetrical thickening of the right upper truncus (arrows). Coronal T1W image (b) shows asymmetrical volume loss (arrow) and fat-suppressed T2W image (c) shows edema (arrow) at the supraspinatus muscle indicating radiological signs of denervation atrophy. TIRM, turbo inversion recovery magnitude; MR, magnetic resonance.

Cervical MRI and MR angiography showed longus colli muscle atrophy, a pseudo-meningocele cavity at the post-foraminal segment of the right C5 nerve, and the absence of the flow in the right VA (Figure 2). Brachial plexus MRI showed the asymmetrical thickening of the upper trunk of the right brachial plexus and compatible findings with denervation atrophy at the supraspinatus muscle (Figure 3). Electroneuromyographic findings were compatible with partial degeneration of the upper trunk in the right brachial plexus. Sensory nerve conduction studies revealed abnormalities in the median sensory nerve of Digit 1 and lateral antebrachial cutaneous sensory nerve on the right side. The electrical stimulation of the brachial plexus at Erb's point showed delayed latency values and the decreased compound muscle action potential amplitude of axillary nerve on the right. Needle electromyography showed chronic neurogenic motor unit changes and reduced recruitment pattern in the right upper trunk muscles (deltoid, biceps, brachioradialis, supraspinatus muscles). No abnormal spontaneous potentials were recorded. Computed tomography angiography confirmed the VA occlusion. The patient was consulted with the Neurology and Interventional Radiology Department and underwent antiplatelet therapy with clopidogrel.

At the end of six months of rehabilitation program, right shoulder abduction, elbow flexion, and forearm supination were 4/5 and the other muscle groups’ strengths were normal. Ptosis of the right eye was slightly recovered and anhidrosis was resolved (Figure 1).

## Discussion

We present a patient with Horner’s syndrome concomitant with VA occlusion and brachial plexus injury that arose after the anterior approach disc herniation surgery.

Horner's syndrome is one of the rare complications of anterior approach cervical disc herniation surgery [7]. Although it is only a minor cosmetic complication, accompanying neurological damage can cause disability. The incidence of Horner’s syndrome after anterior cervical discectomy varies from 0.06 to 0.1% [5,8].

Accidental injury of the sympathetic tract may occur with the anterior approach cervical spine surgery [6]. The prolonged retraction of the longus colli muscle is thought to be responsible for the sympathetic trunk injury [5]. In line with previous reports we observed a pseudo-meningocele cavity at the C5-6 level, reported being the most affected level through the cervical chain [5]. The anatomical proximity of the cervical sympathetic trunk to longus colli muscle makes it vulnerable to injury at the lower part (especially at the C5-6 level) of the cervical spine [6,9]. In our case, electroneuromyographic findings were compatible with brachial plexus upper trunk injury. MRI and computed tomography angiography findings revealed the right VA occlusion, atrophy of the right longus colli, and supraspinatus muscles, in addition to the damage of the brachial plexus at the level of C5, C6 nerve roots.

It is known that VA is vulnerable to injury and occlusion of VA can be seen as a result of cervical fracture and sharp head movements and can occur even with chiropractic manipulation [10]. However, iatrogenic VA injury is a rare complication of anterior approach cervical spine surgery [11]. Paghi et al. reported VA injury at the same level as our report in a young male patient with cervical osteoblastoma who had undergone anterior approach cervical surgery [11]. Yunoki et al. reported that repeated use of a spreader in two consecutive intervertebral space may have triggered VA occlusion after anterior cervical discectomy in a 50-year-old patient [10]. The potential underlying mechanism of VA in issue is intimal disruption followed by thrombus formation causing occlusion of the artery lumen [10,12].

Kim et al. described a case with Horner’s syndrome secondary to VA stenosis and concomitant carotid artery dissection in a patient with orbital and nasal bone fractures and chest tube [13]. We propose that Horner’s syndrome is caused by damage to the sympathetic fibers extended along with longus colli muscle that can be sectioned, cauterized, or retracted as part of the surgical approach. Since VA is not a part of the oculosympathetic pathway, VA stenosis is unlikely the cause of Horner’s syndrome. However, he had no symptoms associated with vertebrobasilar insufficiency. Although the higher number of anterior cervical discectomy and fusion level is a risk factor for developing VA injury, our patient had undergone one-level cervical discectomy and cervical disc prosthesis operation surgery [7]. There have been a little data for the functional recovery of Horner's syndrome that arose after anterior approach spine surgery. In our patient, while ptosis was slightly recovered, anhidrosis was fully resolved. Brachial plexopathy after cervical spine surgery can result from patient positioning or anterior approaches. Moreover, brachial neuritis (Parsonage-Turner syndrome) is another possible cause of brachial plexopathy after cervical spine surgery. Our patient presented with pain in the right shoulder region, proximal upper extremity weakness, and sensory disturbances on C5-6 dermatomes. Since the most important differential diagnosis to be considered in this clinical presentation is C5 root damage, electrophysiological examination is of great importance. Electroneuromyography and nerve conduction studies revealed partial injury of the upper truncus of the right brachial plexus. MRI examination of the brachial plexus showed right brachial plexopathy. In this patient, although brachial plexus and VA occlusion was confirmed by imaging methods and electrophysiological study, the diagnosis of Horner's syndrome was made clinically without pharmacologic confirmation by using topical cocaine 4%, that the diagnostic accuracy and sensitivity are not really reliable [2]. Besides, the diagnosis of Horner's syndrome is mostly dependent on the clinical signs.

## Conclusions

To the best of our knowledge, our report is the first one with these three rare complications in the same patient. Horner’s syndrome is rarely complicated with brachial plexus injury and VA occlusion after anterior approach cervical discectomy. Keeping in mind that these complications may be seen together after anterior approach spine surgery will provide timely intervention for possible life-threatening and functional impairments. The differential diagnosis of these three rare complications and the management of their recovery processes are of great importance. Neurological signs should be evaluated carefully even after successful surgical interventions.
